# Overview of the Therapeutic Potential of Aptamers Targeting Coagulation Factors

**DOI:** 10.3390/ijms22083897

**Published:** 2021-04-09

**Authors:** Max Liu, Khalequz Zaman, Yolanda M. Fortenberry

**Affiliations:** Department of Biology, Case Western Reserve University, Cleveland, OH 44106, USA; mxl809@case.edu (M.L.); kxz91@case.edu (K.Z.)

**Keywords:** aptamers, SELEX, coagulation factors, blood disorders, prothrombin, thrombin, plasminogen activator inhibitor, urokinase-type plasminogen activator, tissue-type plasminogen activator, coagulation factors

## Abstract

Aptamers are single-stranded DNA or RNA sequences that bind target molecules with high specificity and affinity. Aptamers exhibit several notable advantages over protein-based therapeutics. Aptamers are non-immunogenic, easier to synthesize and modify, and can bind targets with greater affinity. Due to these benefits, aptamers are considered a promising therapeutic candidate to treat various conditions, including hematological disorders and cancer. An active area of research involves developing aptamers to target blood coagulation factors. These aptamers have the potential to treat cardiovascular diseases, blood disorders, and cancers. Although no aptamers targeting blood coagulation factors have been approved for clinical use, several aptamers have been evaluated in clinical trials and many more have demonstrated encouraging preclinical results. This review summarized our knowledge of the aptamers targeting proteins involved in coagulation, anticoagulation, fibrinolysis, their extensive applications as therapeutics and diagnostics tools, and the challenges they face for advancing to clinical use.

## 1. Introduction

Aptamers are single-stranded nucleic acids, either DNA or RNA, that bind to their target protein with high affinity and specificity. During the early years of aptamers technology, RNA aptamers were preferred since RNA was assumed to fold in a more functional domain. However, recently, DNA aptamers are becoming more prevalent [1]. Both RNA and DNA aptamers are being developed for various uses, including therapeutics [1]. The high-affinity binding of aptamers is accomplished by folding the aptamer in a three-dimensional structure specific to the target protein. Aptamers were first introduced in 1990 by two independent research groups [2,3]. Tuerk and Gold discovered high-affinity binding nucleic acid molecules to the bacteriophage T4 DNA polymerase using a novel iterative method, termed systemic evolution of ligands by exponential enrichment (SELEX) [2]. Ellington and Szostak, also working on the high affinity binding nucleic acids binding to organic dyes, coined the term aptamer, which comes from Optus (Latin for fit) and meros (Greek for part) [3]. Since then, aptamers directed against a wide array of molecules, ranging from small ions [4] to intact cells [5,6,7], including coagulation proteins such as factor VIIa [8,9], factor IX [10,11,12], and thrombin [13,14,15,16]. In general, aptamers alter the protein function by binding to and inducing a conformational change in the protein, which subsequently disrupts the function. The direct inhibition of protein function by aptamers suggests that they are similar to monoclonal antibodies. The aptamers’ technology has improved significantly over the years, making them a useful molecular tool for diagnostic and therapeutic agents. Additionally, their utility has expanded to other applications, including protein chemistry, biochemistry, drug discovery, cell imaging, food science, therapeutics, cancer biology, and stem cell biology [17,18].

Aptamers are a promising family of molecules that can serve as therapeutics in various diseases such as cardiovascular and cancer [19,20,21,22]. They are similar to monoclonal antibodies and are termed “chemical antibodies.” Aptamers are generated to have high specificity, affinity, and they can be adaptable for adequate bioavailability. Compared to monoclonal antibodies, aptamers are synthesized in vitro and not in an animal. They do not require an immune response. They are smaller, reproducible, and the production time is shorter. Compared to other nucleic acid therapeutics such as antisense oligonucleotides and siRNA, aptamers target intracellular, extracellular, and cell surface molecules.

On the other hand, these nucleic acids target intracellular molecules. A recent review addresses the therapeutic advantages and disadvantages of aptamers vs. other molecules and nucleic acids [23,24]. The ability of aptamers to bind to practically any target protein makes them very attractive. Thus, the question that remains is why are aptamers not advancing as therapeutic targets? There are several potential reasons, as discussed in this review.

Numerous aptamers are in clinical and preclinical trials for treating various diseases [25,26]. However, few are approved for clinical use despite the advantages to using RNA aptamers as therapeutic agents. In general, the advantages are: 1) They exhibit high affinity and specific binding to their target protein, 2) aptamers are non-immunogenic, 3) they are synthesized with modified bases to ensure plasma stability, and 4) their half-life in the plasma increases when they are conjugated to high molecular weight polyethylene glycol or cholesterol esters. However, some of these general advantages have been challenged. In 2004, the US Food and Drug Administration approved the first RNA aptamer for use against age-related macular degeneration [27,28] discovered by NeXstar Pharmaceuticals, termed Macugen (pegaptanib). Macugen is an RNA aptamer that binds to the extracellular domain of vascular endothelial growth factor (VEGF) and behaves as an anti-angiogenic molecule [29]. Macugen is used to treat individuals suffering from age-related macular degeneration. Despite the success of Macugen and the promise of aptamer-based therapeutics, no other aptamers have gained US FDA approval for clinical use. However, several are in the pipeline [30,31]. This lag in advancing aptamers for clinical use is due to challenges such as their stability in circulation and renal clearance, both of which result in a decrease in their half-life [32,33]. However, modifications to the aptamer molecules can potentially eliminate these challenges.

The focus of this review article is to provide a general overview of the aptamers to coagulation, anticoagulation, and fibrinolysis proteins and evaluate their status as future therapeutic molecules and how they are being improved to address the challenges such as stability and renal clearance that is hindering the advancement of aptamers for clinical use.

## 2. Blood Coagulation Pathway

**Figure 1:**
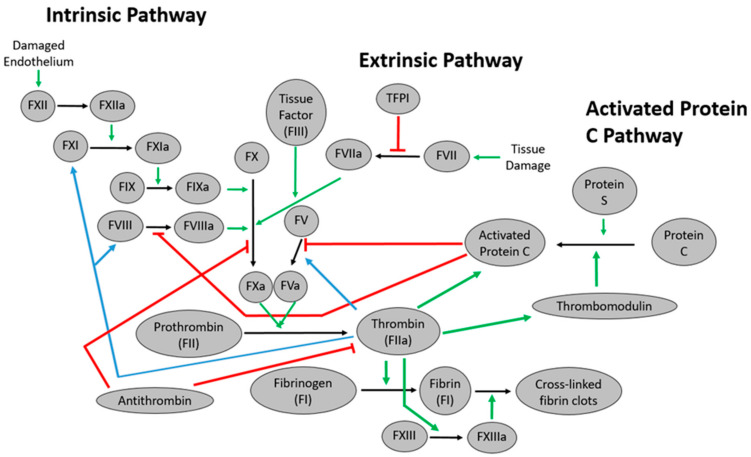
Blood coagulation pathway. Green arrows correspond to the activation of various factors in the blood coagulation pathways; red arrows represent the inhibitory regulation of blood coagulation pathways; and blue arrows show the feedback loop of thrombin.

The ability of blood to “heal” itself is critical to maintaining normal hemostasis. If the blood loses its ability to do this, there are dire circumstances resulting in mortality. Blood is fluid, however, upon vascular injury, blood forms a hemostatic plug, preventing hemorrhaging. When there is a disorder in the normal process of developing and clearing this hemostatic plug, a pathological situation can occur, such as bleeding or thrombosis. Upon injury to the vascular endothelium, platelets adhere to the sub endothelium and become activated, releasing factors and chemicals important in blood coagulation initiation and, ultimately, converting fibrinogen to fibrin. Fibrin stabilizes the initial platelet plug to seal the site of injury. Blood coagulation is a complex pathway cumulating in thrombin, converting fibrinogen to fibrin via the common pathway. Two pathways initiate blood coagulation: the intrinsic and extrinsic pathways (Figure 1).

### 2.1. The Extrinsic Pathway

After the vessel wall injury, platelets adhere to the Von Willebrand factor (vWF) and sub-endothelial collagen. The activation of endothelial cells leads to tissue factor (TF) expression on TF-bearing cells. TF binds to factor VII in circulation, converting factor VII to factor VIIa, forming the TF-VIIa complex (Figure 1). This complex binds to platelets and endothelial cells’ membranes, activating IX and X into factors IXa and Xa. The tissue factor pathway inhibitor (TFPI) partially regulates this pathway (Figure 1). The extrinsic pathway is the predominant activation pathway in vivo, but the intrinsic pathway can also contribute to coagulation.

### 2.2. The Intrinsic Pathway

In the intrinsic pathway, factor XII is activated by prekallikrein and high-molecular-weight kininogen. The intrinsic pathway is triggered when there is an internal injury to the vascular endothelium, exposing endothelium collagen. The intrinsic pathway consists of factors IX, XI, XII, and XIII. The initial step in the intrinsic pathway activates factor XII to factor XIIa, and factor XIIa then aids in activating factor XI to factor XIa. Factor XIa then activates factor IX to factor IXa, leading to the common pathway (Figure 1). Disorders in this pathway lead to disease such as hemophilia.

### 2.3. The Common Pathway

Both pathways lead to the activation of factor X to factor Xa. Factor Xa activates some factor V into factor Va, which then binds to the tissue factor on cell membranes. This complex then recruits factor Xa to form the Xa-Va prothrombinase complex, which converts a limited amount of prothrombin (factor II) into thrombin (Figure 1). While thrombin is crucial for fibrin clot formation, this initial amount of fibrin is not sufficient to form fibrin clots.

Thrombin generated from the prothrombinase complex activates factors VIII and IX into factors VIIIa and IXa, respectively. Factor IXa binds to the activated platelets and forms the tenase complex of factor IXa and VIIIa. The tenase complex augments the activation of factor X into factor Xa, creating a higher amount of prothrombinase complex, which produces enough thrombin for fibrin clot formation. Thrombin also induces the activation of factor XI into factor XIa, which converts more factor IX into factor IXa.

Thrombin activates fibrinogen into fibrin and factor XIII into factor XIIIa. Additionally, thrombin activates protein C, which inactivates factor Va and factor VIIIa. This prevents tenase and prothrombinase from generating more of factor Xa and thrombin, respectively. Thrombin also assists in activating plasminogen into plasmin, which contributes to clot dissolution.

Aptamers have been generated to several factors in all three pathways that are showing promise as potential life-saving therapeutics.

## 3. Extrinsic and Common Pathway Aptamers

### 3.1. Prothrombin/Thrombin Aptamers

**Figure 2:**
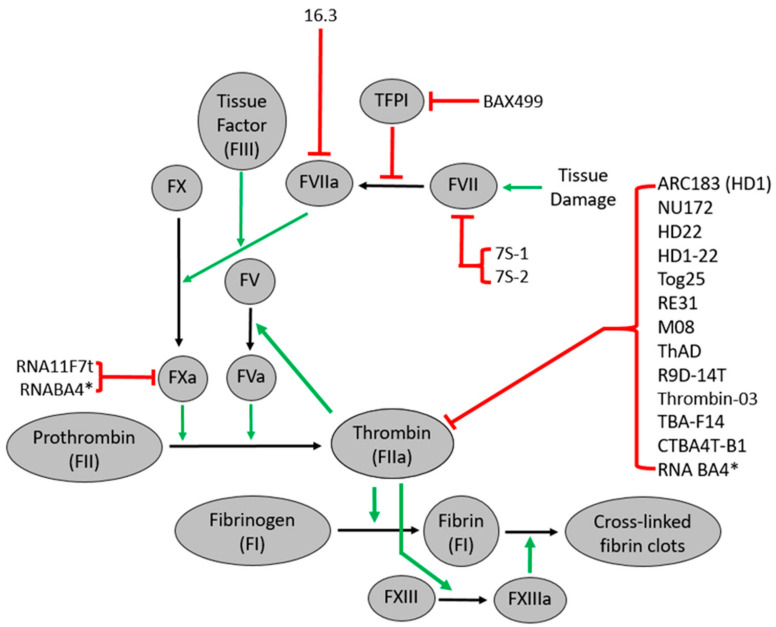
Extrinsic and common pathway aptamers. ARC183 (HD1), NU172, HD22, HD1-22, Tog25, RE31, M08, ThAD, R9D-14T, TBA-F14, and CTBA4T-B1 are aptamers that target thrombin. RNA_11F7t_ decreases clot formation by inhibiting prothrombinase assembly. * RNA_BA_4 is a bivalent made by linking RNA_11F7t_ and R9D-14T and works as an anticoagulant by blocking FXa and thrombin. BAX499 blocks TFPI inhibition of FXa and the TF/FVIIa complex. Aptamer 16.3 promotes anticoagulation by inhibiting FVIIa, while 7S-1 and 7S-2 serve as anticoagulants by inhibiting FVII (Appendix A).

Thrombin has long been a target for anticoagulant therapy due to its central role in the blood coagulation pathway [34]. Thus, direct and indirect thrombin inhibitors are used to treat individuals prone to cardiovascular complications such as acute coronary syndromes, venous thromboembolism, and heparin-induced thrombocytopenia [35,36]. Thrombin cleaves fibrinogen and platelet activated receptors into fibrin and activated platelets, respectively [37,38]. Thrombin stimulates its production by activating factors V and VIII and XI (Figure 1). Thrombin also downregulates itself by activating the protein C pathway. Thrombin contains two binding sites for anions: Exosite I binds to factor V (FV), factor VIII (FVIII), fibrinogen, platelet PAR receptors, and thrombomodulin, while exosite II binds FV, FVIII, heparin, and the platelet receptor GPIbα [37]. Thrombin was one of the first coagulation protein targeted aptamer, as the first reported thrombin-specific RNA was reported in 1992 [13]. Since then, thrombin aptamers (RNA and DNA) have been developed, improved (Figure 2), and continue to be investigated due to their potential as effective potent anticoagulant molecules, as recently reviewed [39,40].

Two thrombin-specific aptamers, HD1 and NU172, have been evaluated in clinical trials. ARC183, also referred to as HD1, is a 15-nucleotide single-stranded DNA aptamer (Archemix Corporation) that binds to prothrombin and thrombin’s exosite I [14]. It inhibits thrombin’s interaction with fibrinogen and competes with factor Va for prothrombin binding [13,41]. HD1 was the first thrombin aptamer to enter phase I clinical trials for an anticoagulant during coronary bypass graft procedures (CABG). HD1 achieved the desired result of the rapid onset of anticoagulation, but the high dose required to initiate anticoagulation resulted in a poor dosing profile [42]. Recent data from whole human blood under flow conditions indicate that HD1 is a potent inhibitor of thrombosis and platelet aggregation [43]. HD1 was modified to incorporate locked nucleic acids, which improved the aptamer’s thermodynamic stability but reduced its antithrombin activity [44]. Esposito et al. changed the sequence of HD1 to develop analogs that reduced lung cancer cells’ proliferation without demonstrating anticoagulant activity [45].

ARC2172 (NU172), developed by ARCA Biopharma and the Nuvelo Corporation, is an unmodified DNA aptamer that binds thrombin’s exosite I [15]. Since NU172 is unmodified, it has a short half-life in vivo, lasting only 10 min in circulation [16]. Compared to ARC183, NU172 is a 1.5-fold more potent anticoagulant [46]. NU172, in a phase Ia study, which enrolled 20 healthy volunteers, exhibited a dose-dependent anticoagulant effect with no adverse side effects [47]. However, the anticoagulant activity was reversed upon removal of NU172 [47], likely due to its short in vivo half-life. NU172 underwent a phase II trial to study its use as an anticoagulant in patients undergoing off-pump CABG surgery. The study was expected to be finished in September of 2013, but its status is currently unknown (NCT00808964).

Considering the challenges associated with the short in vivo half-life, recently, De Fenza et al. incorporated some of the critical NU172 nucleotides (at the T9 and G18 positions) with hexitol-based nucleotides to assess their effect on NU172 anticoagulant activity, binding affinity, and plasma half-life [48]. They found that incorporating these bases slightly improved the NU172 anticoagulant properties. However, there was an increase in binding affinity and substantially greater human serum stability.

Advancing these aptamers to clinical trials require additional preclinical studies to address some of the challenges associated with aptamers in vivo, particularly potency and stability. HD22 is a DNA aptamer developed by NeXstar Pharmaceuticals that binds thrombin’s exosite II [49]. Analyses of the HD22 pharmacological properties conducted in whole human blood under flow conditions revealed that compared to HD1, HD22 exhibits less potent anticoagulant and anti-platelet activity. Surprisingly, HD22 augmented thrombin and fibrin production, likely due to allosteric changes in the thrombin structure [43]. Müller et al. used a poly-dA linker to connect HD1 with HD22 to form the HD1-22 aptamer capable of binding both exosites I and II. They compared HD1-22 with other direct thrombin inhibitors and found that HD1-22 is as potent as bivalirudin and more potent than argatroban [50]. Antidote deoxynucleotides reversed the HD1-22 anticoagulant properties, so HD1-22 has the potential to be used in clinical settings that require anticoagulation and rapid reversal of anticoagulation [50]. Investigators are continuing to improve HD1 and HD22 and assess their pharmacological properties on thrombin formation and platelet aggregation [43].

As with NU172, several groups are attempting to improve upon the pharmacological properties of HD1 and HD22, mainly in vivo stability, by stabilizing the HD1 quadruplex [51,52,53,54]. Varada et al. incorporated a methoxymethyl threofuranosyl thymidine at the T7 position of HD 1, producing more nuclease resistant and thermodynamically stable aptamers that also exhibited a more potent anticoagulant activity [51]. Kovacic et al. introduced pyrene-modified uridine nucleotides to HD1, producing more nuclease resistant aptamers [52]. This modification resulted in a decrease in the aptamer’s thrombin binding affinity. Additional studies have also explored improving the HD1 stability, including removing the 3′and 5′ ends to cyclize HD1, resulting in aptamers with improved stability but reduced potency [55]. Recently, Bao et al. inserted 8-trifluoromethyl-2-deoxyguanosine into HD1, creating the TBA-F14 aptamer, which exhibited improved thermal stability and anticoagulant properties compared to HD1 [53].

In addition to NU172 and HD1, other thrombin-specific aptamers have been developed. However, these molecules have not advanced to clinical trials as of yet. Despite not being tested therapeutically, these molecules have provided valuable information on thrombin’s interaction with aptamers. Tog25 is an RNA aptamer that binds to exosite II of thrombin [56,57]. Tog25 was developed via Toggle SELEX, an in vitro selection method for aptamers where the protein target “toggles” between porcine and human thrombin in alternating selection rounds. As a result of the selection process, Tog25 can bind both porcine and human thrombin with high affinity [56]. Tog25 is less potent than ARC183, but the combination of both thrombin aptamers produced more significant activated partial thromboplastin times, prothrombin times, and thrombin clot times [58].

RE31, a DNA antithrombin aptamer binds exosite I, consisting of a G-quadruplex and a duplex domain linked by four nucleotides [59]. RE31 prevents both the binding of fibrinogen and the thrombin receptor on platelets [59]. Thus, RE31 inhibits fibrin formation and platelet aggregation [59]. RE31 inhibits coagulation more effectively than ARC183 [60]. RE31 analogues with unlocked nucleic acids at the T^15^ position and locked nucleic acids in the duplex part exhibited greater stability. They were twice as potent, suggesting that it is a better potential therapeutic agent [61]. RE31 was recently shown to affect the fibrin cleavage rate by tPA, most likely due to its inhibitory activity towards thrombin-activatable fibrinolysis inhibitor [62].

M08 is a DNA aptamer developed using a microbead-assisted capillary electrophoresis SELEX, with anticoagulant abilities 13-fold stronger than NU172 [46]. A 2-fold-mediated rapid antidote was also designed to ensure that the aptamer can be administered safely, so M08 is another possible anticoagulant/antidote pair aptamer with therapeutic potential [46].

ThAD is a bivalent DNA aptamer that binds both exosites I and II. In vitro analyses demonstrated that ThAD is stronger than argatroban [63,64]. R9D-14T, an RNA aptamer optimized from the previously synthesized R9D-14 aptamer, binds prothrombin and thrombin at pro-exosite I and exosite I, respectively. Since R9D-14T is a more potent anticoagulant than ARC183 and exhibits antidote reversibility, it has the potential to be used when a potent anticoagulant is necessary, such as deep vein thrombosis cardiopulmonary bypass, percutaneous coronary intervention or a stroke [65]. Thrombin-03, also known as Thr-08, is a 100-mer DNA aptamer that binds thrombin with higher affinity by two to three orders of magnitude than ARC183 and HD22. Thr-08 increased clotting times 2-fold compared to ARC183 [66,67].

### 3.2. Factor Xa Aptamers

As with thrombin, factor Xa is central to coagulation and is the target of several anticoagulant therapies. Direct factor Xa inhibitors such as rivaroxaban, apixaban, and edoxaban are used to treat various conditions, including atrial fibrillation, deep vein thrombosis, pulmonary embolism, and stroke [68,69,70,71,72,73,74]. Factor Xa cleaves prothrombin into thrombin, significantly increasing thrombin production when assembled as part of the prothrombinase complex with factor Va, phospholipid membranes, and calcium ions (Figure 1). Two aptamers have been developed to target FXa. RNA_11F7t_ is an RNA aptamer that inhibits prothrombinase assembly (Figure 2), but it does not bind the factor Xa active site [75]. This was the first aptamer targeting FXa [75]. When combined with other factor Xa inhibitors that bind the factor Xa active site, RNA_11F7t_ inhibited clot formation in the human blood moving through an extracorporeal oxygenator circuit that simulated cardiopulmonary bypass surgery [76]. RNA_11F7t_ proved to be as effective as unfractionated heparin, the standard anticoagulant used in cardiopulmonary bypass surgery that is harmful to some patients when combined with a factor Xa inhibitor rivaroxaban, apixaban, and edoxaban [76]. Still, this effect was not observed when only one of these agents was used. There were no differences in endogenous thrombin potential between RNA_11F7t_ and rivaroxaban, apixaban, and edoxaban at the same concentrations. Thromboelastography (TEG) clotting times were higher in samples exposed to RNA_11F7t_ than models exposed to the same rivaroxaban and edoxaban concentrations [76].

RNA_BA_4, a bivalent aptamer produced by covalently linking RNA_11F7t_ and R9D-14T, can bind factor Xa, prothrombin, and thrombin (Figure 2). Complimentary antidote oligonucleotides neutralize RNA_BA_4 effectively. The bivalent aptamer produced the same coagulation level as the two-parent aptamers at the same doses [77]. Despite these aptamers’ promise as anticoagulants, neither RNA_11F7t_ nor RNABA4 has been analyzed in clinical trials.

### 3.3. Tissue Factor Pathway Inhibitor Aptamers

The tissue factor pathway inhibitor (TFPI) negatively regulates the coagulation cascade by inhibiting the complex of factor VIIa, factor Xa, and tissue factor (Figure 1). In addition to N- and C-terminal domains, TFPI has three Kunitz domains: Kunitz 1 (K1) inhibits factor VIIa, Kunitz 2 (K2) inhibits factor Xa, and Kunitz 3 (K3) mediates the TFPI interactions with cell surfaces. Developing anti-TFPI molecules is important in treating hemophilia patients, given the limitations of factor replacement therapy [78]. Consequently, aptamers that inhibit TFPI have been investigated. BAX499 (Baxter Innovations), formerly known as ARC19499, is a modified DNA aptamer that prevents TFPI from inhibiting factor Xa and the TF/FVIIa complex. BAX499 directly interacts with K1, K3, and C-terminal domains of TFPI (Figure 2), but it does not directly compete with FXa [79]. In plasma from hemophilia A and B patients, BAX499 improved thrombin generation and restored clot formation in factor VII-deficient whole blood [80]. Additionally, BAX499 restored clotting and bleeding times in non-human primate models of hemophilia [80]. BAX499 enhanced spatial fibrin formation in plasma from healthy and hemophilia A patients [81]. In an ex vivo study using blood from hemophilia A and B patients, BAX499 produced clotting profiles in hemophiliac blood similar to healthy controls, even for those with more severe hemophilia [82]. While these results suggested that BAX499 is a candidate to treat hemophilia patients, a phase I trial evaluating ARC19499 was terminated due to the decreased TFPI clearance from aptamer binding and raising TFPI levels increased bleeding events [83]. Currently, no other TFPI aptamers are being investigated, most likely due to the improved monoclonal TFPI antibodies with better pharmacokinetic properties than BAX499 [84,85].

### 3.4. Factor VII Aptamers

Factor VII (FVII) and tissue factor (TF) are the extrinsic pathway components for blood coagulation. FVII is activated by binding to TF through protein-protein interactions, forming a complex that allows FVIIa to start FX and FIX, leading to thrombin generation at the injury site. Thrombin can further activate additional FVII via proteolytic cleavage (Figure 1). The modified RNA aptamer 16.3 inhibits TF/FVIIa-mediated activation of FX by preventing TF from complexing with FVIIa [8]. Compared to other FVIIa antagonists, 16.3 is as potent of an anticoagulant in vitro as hTFAA, a mutant form of human tissue factor that inactivates FVIIa. It is 100-fold less potent than KDTF5, a derivative of hTFAA [8]. The RNA aptamer 16.3 has not been analyzed clinically since its secondary structure changes at physiological temperatures, resulting in decreased affinity for FVIIa [8]. Two 2′fluoropyrimidine RNA aptamers named 7S-1 and 7S-2 bind FVIIa, but 7S-1 is a much more potent anticoagulant (Figure 2). At 30 nanomoles, 7S-1 inhibits at least 90% of FVIIa activity in PT clotting assays [9].

## 4. Intrinsic and APC Pathway Aptamers

### 4.1. Factor IXa Aptamers/Antidote Pair

**Figure 3:**
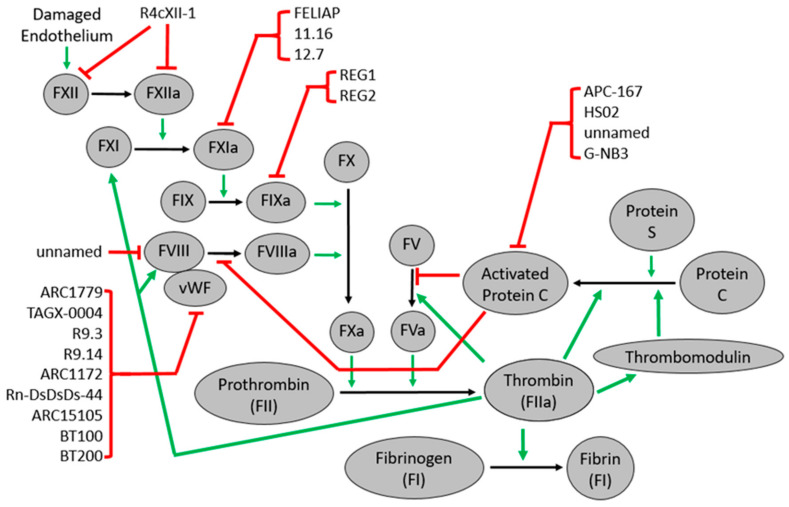
Intrinsic and APC pathway aptamers. R4cXII-1 inhibits FXII and FXIIa, blocking the activation of FXI. FXIa inhibitors REG1 and REG2 are anticoagulants that displayed promising results by inhibiting FIXa. FELIAP, 11.16, and 12.7 decreased the activation of FIX. An unnamed aptamer whose therapeutic potential was not tested binds FVIIIa. ARC1779, TAGX-0004, R9.3, R9.14, ARC1172 Rn-DsDsDs-44, ARC15105, BT100, and BT200 are anti-WF aptamers. APC-167 is a noncompetitive inhibitor of APC. G-NB3, HS02, and an unnamed aptamer promote thrombin generation by inhibiting the APC anticoagulant properties (Appendix A).

Factor IX (FIX) converts to factor IX (FIXa) either from FXIa or TF/FVIIa complex. Once activated, FIXa forms a complex with factor VIIIa, before activating factor X (Figure 1). Aptamers targeting factor IX and factor IXa have been evaluated as anticoagulants in several clinical trials [12,86,87,88]. The modified RNA aptamer, 9.3t, binds the extended substrate-binding site (exosite) of FIX and FIXa. Aptamer 9.3t inhibits FX activation without inhibiting the FVIIIa/FIXa complex [11] (Figure 3). The antidote oligonucleotide, 5-2, which is composed of nucleotides complementary to a portion of the 9.3t aptamer, reverses anticoagulation induced by 9.3t within 10 min in vitro [10]. This groundbreaking study was the first to demonstrate that a complementary oligonucleotide strand can reverse an aptamer’s effects [10]. This anticoagulant-antidote pair of direct factor IXa inhibitor pegnivacogin (RB006) and its antidote anivamersen (RB007) is named REG1, which was developed by Regado Biosciences and tested in patient trials.

REG1 was evaluated in phase I trials in healthy patients and patients with stable coronary artery disease on anti-platelet therapy. In this phase, REG1 was a safe and effective anticoagulant. There were no reported serious side effects or significant bleeding events that resulted from REG1 [12,86]. In a phase II trial with 640 patients, REG1 proved to be an effective anticoagulant that prevents bleeding and thrombotic events in acute coronary syndrome (ACS) [87]. Enrollment in the group receiving 25% reversal was stopped early since three of the 41 patients developed allergic reactions [87].

REG1 progressed into a phase III trial that planned on enrolling 13,200 patients. Despite this novel anticoagulant/antidote treatment’s early success, the phase III trial ended early after 10 patients developed severe allergic reactions, including one fatal event [88]. Given the aptamers’ non-immunogenic property, it was determined that the allergic reactions are believed to be caused by the PEG portion of pegnivacogin, not the aptamer moiety [89]. Patients who experienced allergic reactions had significant preexisting anti-PEG antibody levels [89]. Later analyses revealed that the aptamer’s PEG moieties were targeted by anti-PEG antibodies, inhibiting the aptamer’s anticoagulant activities in vivo and in vitro [90]. There was no evidence that REG1 decreased ischemic events more than bivalirudin, but the study’s statistical power was limited [88]. Although REG1 produced early encouraging results, its adverse effects prevented it from being a practical, helpful treatment option.

Regado Biosciences developed a nearly identical system termed REG2 that was also studied in clinical trials. REG1 and REG2 used the same aptamer and antidote, but REG1 and REG2 were administered intravenously and subcutaneously, respectively. In a phase I trial involving 36 health patients, REG2 proved to be well-tolerated and initiated anticoagulation for several days that could be reversed with the antidote. This trial was the first attempt to study subcutaneously administered aptamers [91]. Despite promising results from the phase I trial, REG2 has not undergone further clinical testing.

Staudacher et al. found that pegnivacogin reduces platelet activation and aggregation in whole blood samples from healthy volunteers, an effect that was reversed with the administration of anivamersen [92]. They observed reduced platelet aggregation in blood samples from ACS patients after intravenous pegnivacogin, suggesting that an aptamer targeting FIXa may be a promising therapeutic for ACS patients [92].

The various trials’ results and outcomes using REG1 exposed aptamers’ limitations as therapeutics: renal clearance and short half-lives [89,91,93]. Adding PEG to the aptamers to increase their molecular weight, thus reducing renal clearance due to size, addressed these challenges. This also results in an increase in its in vivo half-life. However, this common moiety used to prolong in vivo activity proved fatal, especially in patients with anti-PEG antibodies [91]. Consequently, for aptamers to move forward as therapeutic, several investigators explore other ways to increase the in vivo half-life, including chemically modifying the nucleotide bases [32,33,94]. The PEG-associated issue is not limited to aptamers, as other PEGalyated molecules also elicit an in vivo immune response, as recently reviewed (Kozma G et al., 2020). Despite the challenges associated with the PEG moiety, the FDA has approved approximately 20 PEGalayted molecules for clinical use (Kozma G et al., 2020). Some groups are investigating ways to neutralize the PEG moiety (Abu Lila AS et al., 2018; Hoang TT et al., 2020). Regardless of the reason for terminating REG1 clinical trials, the factor IXa aptamers ultimate failure for clinical use dampened the excitement and promise of aptamers as therapeutics. Nonetheless, research in this area continues to improve aptamers as therapeutics [40].

### 4.2. Factor XIa Aptamers

Factor XIa (FXIa) is a serine protease that cleaves FIX, activating it into FIXa (Figure 1). FXIa consists of two identical subunits that contain a catalytic domain and four apple domains named A1 through A4 [95]. The A3 domain has anion-binding sites that are essential for the FXIa activity [96,97]. Developing factor XIa inhibitors as potential anticoagulant agents focus on several patents filed before 2016, however, fewer molecules are developed [98]. A comprehensive review of the various factor XIa inhibitors was recently published [98]. The factor XI inhibiting aptamer (FELIAP) is a DNA aptamer that binds FXIa at or near its active site with high affinity (Figure 3), but it is not a potent anticoagulant [99]. Milimolar concentrations of FELIAP were required to inhibit nanomolar levels of FXIa from activating FIX and forming a complex with antithrombin [99]. However, FELIAP could serve as a lead compound for more potent FXIa-inhibiting aptamers, similar to how monosulphated benzofurans acted as lead compounds for other factor XIa inhibitors [99]. Woodruff et al. developed two RNA aptamers named 11.16 and 12.7 (Figure 3). Both aptamers are non-competitive inhibitors of FXIa by binding FXIa ABS2 and a charged area on the FXIa autolysis loop. While both 11.16 and 12.7 increased the activated partial thromboplastin time (aPTT), a more significant increase in aPTT was observed when 12.7 was administered [100]. There are numerous factor XIa inhibitors that have been evaluated in clinical trials [98]. Despite promising data from some of the factor XIa aptamers, it appears that the more direct inhibitors, such as monoclonal antibodies and small-molecule inhibitors, are more effective than the aptamers. Thus, research in this area has declined but developing these molecules remains relevant.

### 4.3. Factor XII Aptamers

Factor XII (FXII) is activated into factor XIIa (FXIIa) by several anionic compounds. FXIIa activates factor XI to initiate the intrinsic pathway of blood coagulation (Figure 2). FXIIa enhances inflammatory responses by activating plasma kallikrein, which cleaves bradykinin, an inflammatory mediator, from high-molecular-weight kininogen. R4cXII-1 is a modified RNA aptamer and a potent anticoagulant that binds FXII and FXIIa without altering the active site activity [101]. R4cXII-1 blocks FXII auto-activation and activation of FXI, but it does not inhibit FXII-mediated activation of plasma kallikrein. R4cXII-1 prevents anionic substances from activating FXII, but it does not activate FXII itself [101]. R4cXII-1 has not been evaluated in clinical trials (Figure 3).

### 4.4. Factor VIII Aptamers

Factor VIII is bound to the Von Willibrand factor in circulation. It separates from the von Willebrand factor upon activation into factor VIIIa. Factor VIIIa is part of the complex that activates factor X (Figure 2). An unnamed DNA aptamer that binds factor VIII was developed. This aptamer was designed for protein purification purposes only [102]. Currently, no other factor VIII aptamers are being studied.

### 4.5. Activated Protein C Aptamer

Protein C is activated into activated protein C (APC) by the complex of thrombin and thrombomodulin in the presence of protein S. APC is a serine protease with a basic exosite involved in the inactivating factors Va and VIIa (Figure 1). APC protects cells from inflammation and apoptosis through its interactions with the endothelial protein cell receptor (EPCR) and protease-activated receptor-1 (PAR-1). APC-167 is an RNA aptamer that binds APC with high specificity and acts as a non-competitive inhibitor to APC-mediated amidolytic cleavage of substrates [103]. When the primers of APC-167 were deleted, the resulting aptamer named APC-99 exhibited similar binding and inhibitory properties [103]. HS02 is a DNA aptamer that binds the APC basic exosite, inhibiting the APC anticoagulant activity without altering its cytoprotective and antiapoptotic functions. HS02 enhances the APC reactivity with protein C inhibitor [104]. The effects of HS02 can be reversed by the oligonucleotide antidote AD22, suggesting that this pair is a treatment candidate for APC-related bleeding disorders such as trauma-induced coagulopathy [105] (Figure 3).

An unnamed anti-APC aptamer from Archemix Corporation decreased clot times and improved thrombin generation in vitro, indicating that this aptamer inhibits APC’s anticoagulant activity. The anti-APC aptamer did not interfere with the APC interactions with EPCR and PAR-1, so the aptamer did not inhibit the APC cytoprotective functions [106]. Another unnamed APC aptamer with a nanomolar dissociation constant was developed using capillary electrophoresis, but its treatment applications were not evaluated [107]. G-NB3 is an aptamer that binds APC’s basic exosite but does not target similar serine proteases or protein C in its inactive zymogen form. G-NB3 inhibited the inactivation of FVa and FVIIIa without blocking the anti-inflammatory and cytoprotective properties of APC [108]. None of these aptamers against APC have progressed to patient trials. However, G-NB3 seems to be a promising therapeutic candidate.

## 5. Fibrinolysis Aptamers

### 5.1. PAI-1 Aptamers

**Figure 4:**
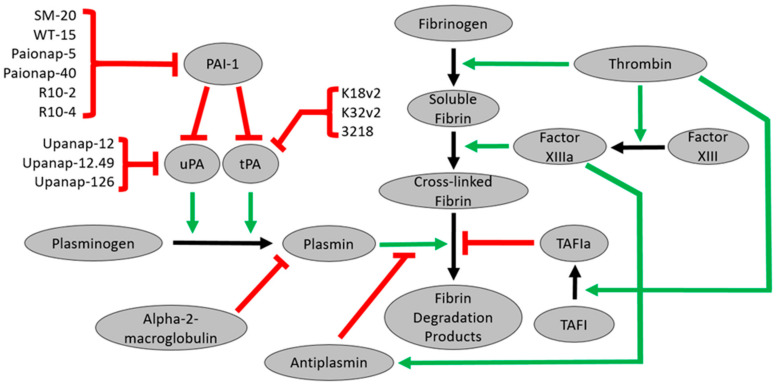
Fibrinolysis pathway aptamers. Paionap-5, paionap-40, SM-20, and WT-15 bind PAI-1, but these aptamers do not alter the PAI-1 antiproteolytic activity. R10-2 and R10-4 inhibit the PAI-1 antiproteolytic against tPA but do not affect PAI-1 interactions with uPA. Upanap-126 inhibits activation of uPA but does not inhibit uPA-mediated plasminogen activation. Upanap-12 and upanap-12.49 inhibit uPA from binding to uPA receptor (uPAR), but neither inhibits uPA catalytic activity. K18v2 and K32v2 are tPA aptamers that inhibited tPA from interacting with LRP-1. The bivalent aptamer 3218 is a potent fibrinolysis inhibitor (Appendix B.

Plasminogen activator inhibitor-1 (PAI-1), a serpin, is the primary inhibitor of the fibrinolysis system. PAI-1 inhibits urokinase-type plasminogen activator (uPA) and tissue-type plasminogen activator (tPA), reducing the activation of plasminogen into plasmin (Figure 4). PAI-1 binds vitronectin on the surface of breast cancer cells, a critical step in metastasis.

Blake et al. developed two aptamers, SM-20 and WT-15, which bind PAI-1 with high specificity and affinity (Figure 4). Both aptamers inhibited the PAI-1 interactions with heparin and vitronectin, but neither aptamer disrupted the PAI-1 antiprotease activity [109]. SM-20 increased cellular adhesion in breast cancer cells by limiting vitronectin detachment from breast cancer cells in the presence of PAI-1 [109]. These results suggested that SM-20 is a promising antimetastatic agent that could be used to treat breast cancer patients [109]. These two aptamers prevented PAI-1 from inhibiting the migration of endothelial and smooth muscle cells, suggesting that they may limit the adverse effects of increased PAI-1 on vascular disease pathogenesis [110]. When expressed endogenously in breast cancer cells, SM-20 and WT-15 inhibited angiogenesis [111].

Paionap-5 and paionap-40 have modified RNA aptamers that bind the flexible joint region of PAI-1 (Figure 4). While paionap-40 only bound native PAI-1, paionap-5 bound both relaxed and stressed conformations of PAI-1. Both aptamers inhibited the PAI-1 interactions with vitronectin, but neither inhibited its antiproteolytic activity [112]. Damare et al. synthesized three RNA aptamers that bind PAI-1. Both R10-2 and R10-4 inhibited the PAI-1 antiproteolytic activity against tPA in a concentration-dependent manner without altering the PAI-1 interactions with uPA [113]. PAI-1-01 is a DNA aptamer capable of binding PAI-1 with high affinity (*K*_d_ of 339 pM). However, its properties as a therapeutic were not evaluated [67]. All of these PAI-1 aptamers remain in the preclinical phase of the study.

### 5.2. The uPA Aptamers

The urokinase-type plasminogen activator (uPA) is a serine protease that activates plasminogen into plasmin on cell surfaces. Pro-uPA is activated into uPA by proteolytic cleavage (Figure 4). The uPA binding to uPA receptor (uPAR) plays a crucial role in metastasis. Dupont et al. found 13 modified RNA aptamers that inhibit uPA–uPAR binding without altering the uPA catalytic activity at low nanomolar concentrations [114]. Five of the six most potent inhibitors had similar predicted secondary structures. Upanap-12, one of the potent inhibitors, was truncated into upanap-12.49, which exhibited the uPA–uPAR inhibitory activity identical to the full-length version (Figure 4). Upanap-12.49 inhibited endocytosis of the uPA–PAI-1 complex and assembly of plasminogen and uPA on the cell surfaces [114]. Upanap-126 is an aptamer derived from RNA that inhibited the activation of uPA but did not inhibit uPA-mediated plasminogen activation [115]. Upanap-126 also inhibited pro-uPA from binding to uPAR and vitronectin to the complex of pro-uPA/uPAR. The aptamer also reduced human tumor cell invasion in vitro and tumor cell intravasation in chick embryo spontaneous metastasis models [115]. Skrypina et al. used SELEX to develop aptamers that bind uPA but did not assess their therapeutic potential [116]. These aptamers have not been evaluated in clinical trials.

### 5.3. The tPA Aptamers

The tissue-type plasminogen activator (tPA) is a serine protease that catalyzes plasminogen conversion into plasmin (Figure 4). In addition to its fibrinolysis role, tPA regulates the blood-brain barrier through its interactions with low-density lipoprotein receptor-related protein-1 (LRP-1). Recombinant tPA is the only treatment for cerebral ischemic stroke but is associated with adverse effects, such as the increased risk of cerebral hemorrhage and neuronal cell death. Two RNA aptamers, K18v2 and K32v2, inhibited tPA from interacting with LRP-1 [117]. In vitro and ex vivo analyses revealed that these two aptamers had negligible impacts on clot lysis. These results suggest that administering these aptamers with tPA to treat cerebral ischemic stroke could reduce the tPA adverse effects interacting with LDL receptors [117]. K18v2 and K32v2 bind to the B-chain and A-chain of tPA, respectively. K18v2 and K32v2 were combined to form a bivalent aptamer named 3218, a more potent fibrinolysis inhibitor than the monomeric aptamer [118]. Despite promising preclinical results, these aptamers have not been evaluated clinically.

## 6. Von Willebrand Factor Aptamers

The Von Willebrand factor (vWF) activates platelets and recruits them to the injury site through an interaction between the A1 domain of vWF and platelet-receptor glycoprotein Ibα (GPIbα). The vWF binds to factor VIII in plasma, making factor VIII more stable. ARC1779 is a vWF-binding aptamer (Archemix Corporation) combined with unmodified DNA, modified 2′-o-methyl-nucleotides, and inverted deoxythymidine [119]. ARC1779 inhibits all vWF-mediated activation pathways and prevents pathological thrombosis by binding to the A1 domain of activated vWF and preventing vWF from interacting with the GPIb receptor on platelets [119] (Figure 3). In a phase I trial with 47 healthy clients, ARC1779 proved to be generally well-tolerated, in which minor adverse events were dose-independent and no bleeding was observed [119].

ARC1779 has been tested to treat several conditions, such as thrombotic thrombocytopenic purpura (TTP), von Willebrand disease type 2B (vWD-2b), and thrombotic microangiopathy (TMA) [120,121,122]. A phase II trial evaluated the use of ARC 1779 to treat two vWF-related platelet function disorders: TTP and vWD-2b (NCT00632242). ARC1779 proved to be a potent vWF inhibitor in vWD-2b patients and prevented platelet count decreases induced by desmopressin [120]. Investigators found that ARC1779 blocked TTP progression in ADAMTS13-deficient patients [121]. Ex vivo studies demonstrated that ARC1779 is a potent and specific inhibitor of vWF, even for acute myocardial infarction patients (AMI) who have an elevated vWF activity [123]. A phase II trial studying ARC1779 in AMI patients was terminated in January 2009 with no results available (NCT00507338). ARC1779 was also examined in a phase II trial for vWD-2b patients, but the study was withdrawn in August 2009 (NCT00694785). ARC1779 was tested for treating thrombotic microangiopathy (TMA) patients in a phase II trial. Like the other trials, the study was terminated in November 2009 due to a slower than expected enrollment (NCT00726544). Data from a limited sample of nine enrolled patients suggested that ARC1779 was a safe method to treat TMA patients [122]. ARC1779 also decreases cerebral embolization after carotid endarterectomy [124]. While investigators found that ARC1779 lowers thromboembolism, they terminated the phase II trial due to the slow enrollment (NCT00742612) [124]. In an ex vivo study, ARC1779 demonstrated similar anti-thrombotic effects to the platelet aggregation inhibitor abciximab in coronary artery disease patients, but ARC1779 was less effective at inhibiting platelet activation and aggregation [125]. ARC1779 also improves refrigerated platelets’ survival and function, suggesting that a short-acting inhibitor of the vWF interaction with platelets may improve refrigeration of platelets for transfusions [126].

ARC15105 (Archemix Corporation) inhibited platelet adhesion on denuded porcine aortas at levels similar to abciximab [127]. Zhu et al. added four nucleotides to improve the stability of ARC15105, producing an aptamer named BT100 [128]. Then, they pegylated BT100 to produce BT200, which is capable of binding human vWF [128]. By binding to the A1 domain of vWF, BT200 inhibits the activities of the A1 domain. In cynomolgus monkeys, BT200 inhibited the platelet function and prolonged bleeding time [128]. This group also developed BT101, a complimentary aptamer that effectively reversed BT200 [129]. Ex vivo analyses conducted in blood from ACS patients revealed that BT200 inhibited platelet plug formation and vWF activity [130]. The promising preclinical data from these aptamers warrants further evaluation in patient trials, with a phase I trial analyzing BT200 in patients with various types of blood disorders that began in December 2020 (NCT04677803).

A recent study shows that in blood samples from patients with large artery atherosclerosis stroke (LAA), BT200 inhibits vWF and platelet function. To prevent a secondary stroke in LAA patients, antiplatelet drugs are administered to the patient. This study demonstrated that the BT200 activity is not altered by antiplatelet drugs, suggesting that BT200 is a potential therapeutic in LAA patients [131].

Several other vWF aptamers have been developed but not tested clinically. TAGX-0004 is a DNA aptamer that demonstrated significant inhibition of thrombus formation compared to ARC1779 [132]. TAGX-0004 displayed a better pharmacological profile and similar inhibitory properties to caplacizumab, a nanobody used to treat acquired thrombotic thrombocytopenic purpura (aTTP), suggesting that TAGX-0004 is a solid candidate to treat patients with aTTP [132]. Oney et al. developed two RNA aptamers, R9.3 and R9.14, capable of binding vWF. Both aptamers’ concentrations greater than 40 nM completely inhibited platelet plug formation in PFA-100, mimicking the platelet function in whole blood under high shear stress [133]. The oligonucleotide antidote AO6 reversed the R9.14 anti-platelet activity within 2 min [133]. ARC1172, the aptamer made by Archemix Corporation that led to the development of ARC1779, prevents vWF from interacting with GPIbα by binding the vWF A1 domain [119,134]. DTRI-031 is an RNA anti-vWF aptamer that inhibits thrombosis in mice and platelet aggregation in whole blood [135]. An antidote oligonucleotide rapidly reversed the DTRI-031 activity [135]. Matsunaga et al. found several DNA aptamers that bind vWF, including Rn-DsDsDs-44, which binds vWF with a high affinity [136]. The aptamer was optimized by adding mini-hairpin loops, which improved the aptamer’s stability without affecting its association for vWF [136].

## 7. Conclusions

Over the past several years, more aptamers are being designed as potential therapeutics to treat patients with various disorders. Since cardiovascular disease is the second leading cause of death worldwide, preventing this disease or treating patients prone to this disease is of utmost importance. Despite the benefit of using aptamers as a therapeutic, there are several drawbacks such as large-scale synthesis, cost, pharmacokinetics, and two of the most relevant ones are shortened half-life and renal clearance. When comparing aptamers to antibodies or small molecule inhibitors, one must consider the advantages of aptamers, including their versatility, ability to target an array of molecules, and low immunogenicity and toxicity [137]. While few aptamers have reached FDA approval for treating patients, aptamers can revolutionize therapeutic medicine with new and improved technologies. Currently, several groups are investigating ways to improve upon the aptamer’s half-life and renal clearance. Circular DNA aptamers can overcome some of these challenges [55,138]. New circular DNA aptamer CTBA4T-B1, binds to exosite 1 of thrombin. This aptamer is a potent anticoagulant, binds with a high affinity to thrombin, and most importantly, it has a half-life of approximately 8 h in the human serum [138]. Other ways to improve the half-life have been investigated as well, including adding modified bases [32,51]. Can aptamers’ uniqueness overcome the disadvantages prompting researchers to continue developing aptamers as therapeutic agents? The answer to these questions remains under investigation. However, it appears to be yes, as more improved aptamers are being developed, which gives us hope that aptamers will be used for clinical use. Aptamers are also being used in conjunction with other known agents such as siRNA and direct coagulation inhibitors, and areas of research that could potentially be the future of aptamers technology. The future success of aptamers as therapeutics hinges on overcoming the in vivo challenges. As research continues in this field, focusing on addressing these challenges and developing new improved aptamers molecules, the promise of these molecules as therapeutics remain. Thus, aptamer-based therapeutics in coagulation or other blood-related disease, such as sickle cell disease [139], remain important to the future of nucleic acid based medicine.

## Data Availability

Data sharing not applicable. No new data were created or analyzed in this study. Data sharing is not applicable to this article.

## Appendix A: List of Aptamers at Various Pre-Clinical Stages.

**Aptamer**	**Type**	**Target**	**Stage**	**References**
ARC183 (HD1)	DNA	Thrombin (exosite 1)	Phase I	[13,14,41,42,43]
NU172	DNA	Thrombin (exosite 1)	Phase II	[15,16,46,47]; NCT00808964
HD22	DNA	Thrombin (exosite 2)	Pre-clinical	[43,49]
HD1-22	DNA	Thrombin exosite 1 and 2)	Pre-clinical	[50]
TBA-F14	DNA	Thrombin (exosite 1)	Pre-clinical	[53]
Tog25	RNA	Thrombin (exosite 2)	Pre-clinical	[56,57,58]
RE31	DNA	Thrombin (exosite 1)	Pre-clinical	[59,60,61]
M08	DNA	Thrombin	Pre-clinical	[46]
ThAD	DNA	Thrombin (exosites 1 and 2)	Pre-clinical	[63,64]
CTBA4T-B1	DNA	Thrombin (exosite 1)	Pre-clinical	[138]
R9D-14T	RNA	Prothrombin and Thrombin (pro-exosite 1 and exosite 1)	Pre-clinical	[65]
Thrombin-03	DNA	Thrombin	Preclinical	[66,67]
RNA11F7t	RNA	Factor Xa	Pre-clinical	[75,76]
RNABA4	RNA	Factor Xa, Prothrombin, and Thrombin	Pre-clinical	[77]
REG1	RNA	Factor IX and IXa (exosite)	Phase III	[10,11,86,87,88,89,90]; NCT00113997, NCT00715455, NCT00932100, NCT01848106
REG2	RNA	Factor IX and IXa	Phase I	[91]
FELIAP	DNA	Factor XIa	Pre-clinical	[99]
11.16	RNA	Factor XIa	Pre-clinical	[100]
12.7	RNA	Factor XIa	Pre-clinical	[100]
16.3	RNA	Factor VIIa	Pre-clinical	[8]
7S-1	RNA	Factor VII	Pre-clinical	[9]
7S-2	RNA	Factor VII	Pre-clinical	[9]
unnamed	DNA	Factor VIII	Pre-clinical	[102]
ARC1779	DNA	Von Willebrand Factor	Phase II	[119,120,121,122,123,124,125,126]; NCT00507338, NCT00694785; NCT00726544; NCT00742612
TAGX-0004	DNA	Von Willebrand Factor	Pre-clinical	[132]
R9.3	RNA	Von Willebrand Factor	Pre-clinical	[133]
R9.14	RNA	Von Willebrand Factor	Pre-clinical	[133]
ARC1172	RNA	Von Willebrand Factor	Pre-clinical	[119,134]
DTRI-031	RNA	Von Willebrand Factor	Pre-clinical	[135]
Rn-DsDsDs-44	DNA	Von Willebrand Factor	Pre-clinical	[136]
ARC15105	RNA	Von Willebrand Factor	Pre-clinical	[127]
BT100	RNA	Von Willebrand Factor	Pre-clinical	[128]
BT200	RNA	Von Willebrand Factor	Pre-clinical	[129,130,131]; (NCT04677803)
R4cXII-1	RNA	Factor XIIa	Pre-clinical	[101]
BAX499	DNA	Tissue Factor Pathway Inhibitor	Phase I	[79,80,81,82,83]
ARC-167	RNA	Activated Protein C	Pre-clinical	[103]
HS02	DNA	Activated Protein C	Pre-clinical	[104,105]
unnamed		Activated Protein C	Pre-clinical	[106]
unnamed	DNA	Activated Protein C	Pre-clinical	[107]
G-NB3	DNA	Activated Protein C	Pre-clinical	[108]

## Appendix B

**Aptamer**	**Type**	**Target**	**Stage**	**References**
SM-20	RNA	Plasminogen activator inhibitor-1	Pre-clinical	[109,110,111]
WT-15	RNA	Plasminogen activator inhibitor-1	Pre-clinical	[109,110,111]
Paionap-5	RNA	Plasminogen activator inhibitor-1	Pre-clinical	[112]
Paionap-40	RNA	Plasminogen activator inhibitor-1	Pre-clinical	[112]
R10-2	RNA	Plasminogen activator inhibitor-1	Pre-clinical	[113]
R10-4	RNA	Plasminogen activator inhibitor-1	Pre-clinical	[113]
Upanap-12	RNA	Urokinase-type plasminogen activator	Pre-clinical	[114]
Upanap-12.49	RNA	Urokinase-type plasminogen activator	Pre-clinical	[114]
Upanap-126	RNA	Urokinase-type plasminogen activator	Pre-clinical	[115]
unnamed	RNA	Urokinase-type plasminogen activator	Pre-clinical	[116]
K18v2	RNA	Tissue-type plasminogen activator	Pre-clinical	[117]
K32v2	RNA	Tissue-type plasminogen activator	Pre-clinical	[117]
3218	RNA	Tissue-type plasminogen activator	Pre-clinical	[118]
